# Evaluation of peripheral and intranigral AAV-mediated Irisin delivery in a chronic AAV-A53T α-Synuclein overexpression mouse model of Parkinson’s disease

**DOI:** 10.3389/fnagi.2026.1918326

**Published:** 2026-09-28

**Authors:** Chunyan Shu, Jiehong Huang, Wandi Pu, Haoyuan Liang, Zucai Xu, Xijin Wang, Lixi Li

**Affiliations:** 1 Department of Neurology, Tongji Hospital, School of Medicine, Tongji University, Shanghai, China; 2 Department of Neurology, Affiliated Hospital of Zunyi Medical University, Zunyi, China; 3 Department of Neurology and Neurological Rehabilitation, Shanghai Disabled Persons’ Federation Key Laboratory of Intelligent Rehabilitation Assistive Devices and Technologies, Yangzhi Rehabilitation Hospital (Shanghai Sunshine Rehabilitation Center), School of Medicine, Tongji University, Shanghai, China; 4 Department of Biomedical Engineering, Johns Hopkins University, Baltimore, MD, United States

**Keywords:** AAV9-A53T, Irisin, neuroprotection, Parkinson’s disease, α-Synuclein

## Abstract

**Background:**

Parkinson’s disease (PD) is characterized by progressive loss of dopaminergic neurons in the substantia nigra and pathological aggregation of α-Synuclein (α-Syn). Although Irisin has shown neuroprotective effects in acute MPTP-induced and α-Syn preformed fibrils (PFFs) propagation models, its therapeutic effects in a chronic α-Syn overexpression model remain incompletely defined.

**Methods:**

In the present study, we evaluated the effects of AAV8-Irisin delivered either peripherally via tail vein injection or centrally via direct intranigral co-administration in the AAV9-A53T mouse model.

**Results:**

Under the tested doses, routes, and intervention schedule, neither peripheral nor intranigral AAV8-Irisin delivery produced statistically significant improvements in behavioral performance, dopaminergic neuron survival, α-Synuclein pathology, glial activation, or AQP4 polarization; however, numerical trends were observed in select measures, including motor coordination and TH-positive neuron counts.

**Conclusion:**

These findings suggest that, under the current experimental conditions, a clear neuroprotective benefit of Irisin was not readily detectable in this model. Whether modest effects might emerge under different exposure levels, intervention windows, or adequately powered designs remains unknown.

## 1 Introduction

Parkinson’s disease (PD) is the second most common neurodegenerative disorder worldwide, and its prevalence continues to rise with age. Currently, more than 6 million people are affected by PD globally (Li et al., 2025). The core pathological features of PD include progressive loss of dopaminergic neurons in the substantia nigra pars compacta, degeneration of dopaminergic nerve terminals in the striatum, and the formation of Lewy bodies composed primarily of pathological α-Synuclein aggregates (Ye et al., 2023). To date, no disease-modifying therapy has been established that can effectively slow or halt disease progression in clinical practice (Koros et al., 2026). Thus, efforts to identify intervention targets with neuroprotective properties and to validate their efficacy in PD models remain essential for advancing disease-modifying strategies.

Extensive evidence indicates that the central nervous system (CNS) is a key target organ for peripheral hormones and myokines. These soluble signaling molecules participate in regulating neuronal survival, synaptic plasticity, and motor and cognitive functions through the periphery-central communication network (Pedersen, 2019). Epidemiological studies have shown that physical activity is among the most effective non-pharmacological interventions for delaying the progression of neurodegenerative diseases (Gilby et al., 2025; Süß et al., 2026). This suggests that exercise-induced myokines may play a critical protective role in delaying disease progression. Irisin is a recently discovered exercise-induced myokine. It is released into the circulation after proteolytic cleavage of its transmembrane precursor protein, fibronectin type III domain-containing protein 5 (FNDC5), in skeletal muscle (Boström et al., 2012; Wrann et al., 2013). FNDC5 expression is transcriptionally regulated by peroxisome proliferator-activated receptor gamma coactivator 1-alpha (PGC-1α) (Wrann et al., 2013). Its cleavage product, Irisin, not only promotes adipose tissue browning and thermogenesis but also crosses the blood-brain barrier to enter the CNS (Dai et al., 2025; Kam et al., 2022). Within the CNS, it has been implicated in exerting neuroprotective effects on the nigrostriatal pathway (Cai et al., 2026; Zhu et al., 2025).

In PD-related studies, Irisin has been shown to mediate protective effects through multiple mechanisms. These include activating the SIRT1 signaling pathway to restore mitochondrial function, enhancing autophagy flux to promote clearance of pathological α-Syn, and inhibiting NLRP3 inflammasome activation and suppressing NF-κB signaling to reduce neuroinflammation (Ali et al., 2024; Cai et al., 2025; Zhu et al., 2025). In the 1-methyl-4-phenyl-1,2,3,6-tetrahydropyridine (MPTP)-induced acute PD mouse model, Irisin treatment markedly ameliorated motor deficits and preserved tyrosine hydroxylase (TH)-positive neurons in the substantia nigra (Cai et al., 2025; Zhang et al., 2023). Furthermore, in the pathological propagation model triggered by α-Syn preformed fibrils (PFFs), Irisin attenuated pathological α-Syn aggregation and prevented dopaminergic neuron loss (Kam et al., 2022). Collectively, these findings suggest that the FNDC5/Irisin signaling axis may play an important neuroprotective role in PD progression.

However, current studies have predominantly focused on MPTP-induced acute toxicity models (Cai et al., 2025, 2026) or the PFFs injection-based propagation model (Kam et al., 2022; Zhu et al., 2025). Whether Irisin confers comparable protection in a chronic, progressive α-Syn overexpression paradigm remains to be systematically evaluated. Accordingly, the present study employs an AAV-A53T-mediated chronic α-Syn overexpression mouse model (Ip et al., 2017). AAV8-Irisin was delivered via two distinct routes: systemic delivery through peripheral tail vein injection and direct intranigral stereotaxic injection. We systematically assessed its effects on motor behavioral performance, dopaminergic neuron survival, α-Syn pathological deposition, glial activation status, and the polarized distribution of aquaporin-4 (AQP4). The aim was to evaluate whether the two delivery routes produce detectable effects within the AAV-A53T model, thereby contributing to a more comprehensive experimental foundation for evaluating the translational feasibility of Irisin as a therapeutic target for PD.

## 2 Materials and methods

### 2.1 Experimental animals

Male C57BL/6J mice (12 weeks old) were obtained from Beijing Vital River Laboratory Animal Technology Co., Ltd., and used as the subjects in this study. The animals were housed in a specific pathogen-free (SPF) environment equipped with a ventilation system. Mice were maintained on a 12 h light/dark cycle. All animal procedures were conducted in accordance with the National Institutes of Health guidelines for the care and use of laboratory animals and were approved by the Ethics Committee of Tongji University.

### 2.2 Generation of the A53T mouse model by stereotaxic injection of AAV9

Recombinant adeno-associated virus serotype 9 (AAV9) vectors expressing either human SNCA (A53T) with a Flag tag (AAV9-A53T) or GFP (AAV9-GFP) were constructed and packaged by WZ Biosciences Inc., (Shandong, China). The titers were 5.03 × 10^13^ vg/mL for the AAV9-A53T and 3.61 × 10^13^ vg/mL for the AAV9-GFP control. For stereotaxic injection, male C57BL/6J mice aged 12 weeks were anesthetized with isoflurane delivered in air. Anesthesia was induced at 5.0% and maintained at 1.0%–3.0% via a nose cone at a flow rate of 0.35 L/min. The mice were then secured in a stereotaxic frame (68018; RWD Life Science Co., Ltd., Shenzhen, China). Anesthetic depth was monitored based on respiratory rate and reflex responses throughout the procedure. A microinjector was used to deliver 2.0 μL of either AAV9-A53T or AAV9-GFP unilaterally (both adjusted to a concentration of 0.5 × 10^13^ vg/mL) into the right substantia nigra (SN) at a constant rate of 0.2 μL/min. The injection coordinates relative to bregma were as follows: anterior–posterior (AP), −3.0 mm; medial–lateral (ML), −1.3 mm; dorsal–ventral (DV), −4.5 mm, according to the mouse brain atlas of Paxinos and Franklin (Second Edition, 2001).

### 2.3 Tail vein injection of AAV

The recombinant adeno-associated virus serotype 8 (AAV8) vector encoding mouse Irisin was constructed as previously described (Islam et al., 2021). The expression cassette contained the cytomegalovirus (CMV) promoter, a beta-globin intron, the coding sequence of mouse Irisin (mouse FNDC5 ORF 1-140, containing the N-terminal signal peptide and the Irisin domain) fused with a FLAG tag, and an SV40 polyadenylation signal. The virus was packaged into the AAV8 serotype and purified to a final titer of 2.62 × 10^13^ vg/mL (AAV8-Irisin). The control virus, expressing GFP from the same backbone, was prepared under identical conditions and yielded a final titer of 1.19 × 10^13^ vg/mL (AAV8-GFP). These AAV vectors were generated and purified by GeneChem (Shanghai, China). For tail vein injection, AAV aliquots were removed from −80 °C storage, thawed slowly on ice, and diluted to the desired concentration. Each mouse was placed in a restraint device with the tail fully exposed. A volume of 200 μL of the viral solution containing either 1 × 10^10^ vg or 1 × 10^11^ vg per mouse was injected slowly. After injection, each mouse was returned to its home cage and observed for 15–30 min to confirm the absence of obvious abnormalities.

### 2.4 Behavioral tests

Behavioral assessments included the open field test, rotarod test, and pole test. In the open field test, the apparatus consisted of a standard open-field chamber (40 cm × 40 cm × 30 cm). At the beginning of the test, each mouse was placed in the center of the chamber, and a camera recorded its spontaneous locomotor activity for 15 min. The total distance traveled, the immobility time, and the distance traveled in the central area were analyzed using Tracking Master v4.0 software. For the rotarod test, mice first underwent three training sessions with the following speed protocols: constant speed at 10 rpm, constant acceleration from 2 to 20 rpm, and constant acceleration from 4 to 40 rpm. Subsequently, mice were subjected to a 5-min rotarod trial under a constant-acceleration paradigm ranging from 4 to 40 rpm. Each trial was repeated three times, with a minimum inter-trial interval of 30 min. In the pole test, mice were trained to climb down a vertical pole (50 cm in height, 0.5 cm in diameter). The descent time from the top to the bottom was recorded across three repetitions, allowing at least 15 min between successive trials.

### 2.5 Tissue preparation

At the experimental endpoint, the 11 mice per group in the peripheral-treatment cohort were randomly allocated, independently of behavioral outcomes, to histological analysis (*n* = 6 per group) or biochemical analysis (*n* = 5 per group). For protein analyses, mice were euthanized by cervical dislocation. Brain tissues were subsequently collected, and the striatum was dissected under a stereomicroscope. The dissected tissues were rapidly frozen in liquid nitrogen and then stored at −80 °C. For immunofluorescence staining, mice were anesthetized with sodium pentobarbital and transcardially perfused with 1 × PBS to eliminate blood, followed by fixation with 4% paraformaldehyde (PFA) via perfusion. Brains were then harvested, dehydrated through a sucrose gradient, embedded in OCT compound, and stored at −80 °C.

### 2.6 Western blotting

Brain tissues were lysed using RIPA lysis buffer (Beyotime, P0013B) supplemented with a protease inhibitor cocktail (Roche, 4693116001) and a phosphatase inhibitor cocktail (Roche, 4906845001). Total protein concentration was determined using a BCA assay kit (Beyotime, P0010). Equal amounts of protein were separated by SDS-PAGE and transferred onto PVDF membranes. The membranes were blocked with 5% non-fat milk for 1 h at room temperature and subsequently incubated with specific primary antibodies overnight at 4 °C. The primary antibodies used were as follows: anti-TH (Merck, T1299; 1:10000), and anti-GAPDH (Proteintech, 81640-5-RR; 1:10000). After three washes with 1 × TBST, the membranes were incubated with secondary antibodies for 2 h at room temperature. Protein bands were visualized using an ECL protein blotting substrate kit. Band intensities were quantified using ImageJ and normalized to GAPDH.

### 2.7 Immunofluorescence staining and quantification

Brain sections (30-μm-thick) were prepared from frozen tissue for immunofluorescence analysis. Sections containing the substantia nigra and striatum were rinsed three times with 1 × PBS (10 min per wash) and subsequently blocked for 1 h at room temperature in a solution consisting of 5% bovine serum albumin (BSA) and 0.3% Triton X-100 diluted in PBS. Following blocking, the sections were separately incubated overnight at 4 °C with the following primary antibodies: anti-TH (Abcam, ab76442; 1:1000), anti-α-Synuclein (phospho S129) (Abcam, ab51253; 1:1000), anti-AQP4 (Cell Signaling Technology, 59678; 1:1000), and anti-Iba1 (Wako, 019-19741; 1:500). After three additional washes with 1 × PBS, the sections were incubated with appropriate secondary antibodies for 2 h at room temperature. The sections were then washed three more times with 1 × PBS and coverslipped using mounting medium. Fluorescence images were acquired using an upright fluorescence microscope (Olympus BX53).

For quantification of TH-positive cells in the substantia nigra, every sixth section (30-μm-thick) throughout the entire SN was collected from both the injected (right) and non-injected (left) hemispheres. TH-positive cells were counted manually using ImageJ, and the total number of neurons in each hemisphere was estimated by multiplying the summed cell counts by six. For each mouse, the ratio of TH-positive cells on the injected side relative to the non-injected side was calculated and used for statistical comparisons. For assessment of striatal TH fiber density, three coronal sections spanning the central striatum were selected from each mouse. Both the striatum ipsilateral to the injection site and the contralateral striatum were imaged under identical exposure conditions. The entire striatal area in each section was outlined as the region of interest (ROI), and the mean fluorescence intensity of TH immunoreactivity was measured after background subtraction using ImageJ. The fiber density ratio (ipsilateral/contralateral) was then calculated for each animal as an index of striatal TH innervation and used for group comparisons. For quantification of Iba1, GFAP, and phospho-S129 α-Synuclein (p-α-Syn) immunoreactivity in the AAV-injected hemisphere, every sixth section (30-μm-thick) was collected. For Iba1 and GFAP, the SN region was outlined as the ROI based on anatomical landmarks. For p-α-Syn, the ROI encompassed the SN and its adjacent regions to capture the full spatial extent of p-α-Syn pathology. The mean fluorescence intensity of each marker was measured for each section after background subtraction. The values from all sampled sections were averaged to obtain a single mean intensity value per mouse for group comparisons. All immunofluorescence images were captured under identical exposure settings.

### 2.8 Sholl analysis of astrocytes

For morphological analysis of astrocytes, a total of 10–12 astrocytes were randomly selected and analyzed per mouse (*n* = 6 mice per group). Brain sections (30-μm-thick) were prepared from frozen tissue. Sections containing the substantia nigra were processed for immunofluorescence using an anti-Glial Fibrillary Acidic Protein (GFAP)-Cy3 antibody (Sigma, C9205; 1:500) as described above. Morphological analysis of astrocytes was performed using ImageJ. Briefly, astrocytes were skeletonized using the NeuronJ plugin, which was originally designed for neuronal tracing and adapted for astrocytic process reconstruction. Subsequent Sholl analysis was conducted on the skeletonized astrocytic arbors. In this analysis, a series of concentric circles with progressively increasing radii was centered on the soma of each astrocyte, and the number of intersections between GFAP-positive processes and each circle was quantified to evaluate astrocytic complexity. The resulting Sholl curve yielded two key parameters: the peak value, which represents the maximum number of branching points along the processes, and the radius at which this peak occurs, indicating the process length corresponding to the maximal branching complexity. Higher values of both parameters indicate greater morphological complexity in astrocytes.

### 2.9 Analysis of AQP4 polarization

Aquaporin-4 polarization was evaluated as previously described (Sun et al., 2024). For quantification, 50 μm segments centered on blood vessels were analyzed using the line-plot tool in ImageJ software. To calculate the polarity index, a baseline was set to the mean fluorescence intensity within a 10 μm interval spanning −20 μm to −10 μm relative to the peak intensity. Adjusted peak fluorescence values were normalized to the control group means for statistical comparisons.

### 2.10 Co-injection of AAV9-A53T and AAV8-Irisin into the substantia nigra

Male C57BL/6J mice (12 weeks old, *n* = 7 mice per group) received a unilateral stereotaxic injection of viral mixtures into the right substantia nigra as described in Section “2.2 Generation of the A53T mouse model by stereotaxic injection of AAV9.” The animals were divided into four groups receiving the following combinations: (1) AAV9-GFP + AAV8-GFP, (2) AAV9-GFP + AAV8-Irisin, (3) AAV9-A53T + AAV8-GFP, and (4) AAV9-A53T + AAV8-Irisin. Each viral solution was adjusted to a titer of 1 × 10^13^ vg/mL, and 1 μL of each virus was aspirated and mixed, resulting in a total injection volume of 2 μL per mouse. Following viral injection, motor performance was assessed every 4 weeks using the rotarod test as described in Section “2.4 Behavioral tests.” By design, this cohort was intended for the focused assessment of motor behavior and nigral dopaminergic neuron counts; accordingly, glial activation markers and AQP4 polarization were not evaluated in these animals.

### 2.11 Statistical analysis

Blinding was applied during image quantification and behavioral data analysis whenever feasible. GraphPad Prism V.10 was used for all statistical analyses. Data are presented as mean ± standard error of the mean (SEM). Comparisons between two groups were conducted using an unpaired two-tailed Student’s *t*-test. For comparisons involving more than two groups, the assumption of equal variances was first assessed using Bartlett’s test. When equal variances were assumed (Bartlett’s test, *P* ≥ 0.05), standard one-way ANOVA followed by Dunnett’s multiple comparisons test with the A53T + GFP group serving as the single control column was applied. When unequal variances were detected (Bartlett’s test, *P* < 0.05), the Brown-Forsythe and Welch ANOVA was applied, followed by Dunnett’s T3 multiple comparisons test with the A53T + GFP group serving as the control column. For longitudinal behavioral assessments, two-way repeated-measures ANOVA was used, followed by Šídák’s multiple comparisons test to compare treatment groups against the A53T + GFP group at each time point. For the 2 × 2 factorial design (A53T × Irisin) of the intranigral co-injection cohort, two-way ANOVA followed by Tukey’s multiple comparisons test was applied. Statistical significance was indicated as follows: *p < 0.05, **p < 0.01, ***p < 0.001, and ****p < 0.0001.

## 3 Results

### 3.1 AAV9-A53T-injected mice exhibited motor dysfunction and loss of dopaminergic neurons in the substantia nigra

To evaluate the suitability of α-Synuclein overexpression mice as a PD model for assessing Irisin-mediated neuroprotection *in vivo*, the present study established a model via stereotaxic injection of AAV9-A53T into the right substantia nigra, with AAV-GFP-injected animals serving as the control group. At 8 weeks post-injection, the emergence of a PD-like motor phenotype was first examined. In the open field test, A53T mice exhibited a marked reduction in total distance traveled and a pronounced increase in immobility time relative to GFP controls (Figures 1A–C). However, time spent in central area was comparable between GFP and A53T mice (Figure 1D). In the rotarod test, A53T mice showed a significantly shorter latency to fall (Figure 1E), whereas in the pole test, they showed prolonged descent times (Figure 1F). To investigate corresponding central nervous system pathology, brain tissues from A53T and GFP control mice were subjected to immunofluorescence staining. Phosphorylated α-Synuclein-positive cells were detected in the substantia nigra, with immunoreactivity extending into the immediately adjacent ventral tegmental area (VTA), consistent with the anatomical proximity of these regions. In contrast, no appreciable phosphorylated α-Synuclein signal was observed in the striatum of A53T mice (Figure 1G), despite the presence of exogenous α-Synuclein expression in this area (Supplementary Figure 1). Concurrently, A53T mice exhibited a significant loss of tyrosine hydroxylase (TH)-positive neurons in the injected substantia nigra, accompanied by diminished mean fluorescence intensity of TH-positive fibers in the striatum compared to GFP controls (Figures 1H–J). These findings indicate that the A53T model reproduces key nigrostriatal pathological and motor features relevant to PD, including α-Synuclein accumulation, dopaminergic neuron loss, striatal terminal degeneration, and motor impairment.

**FIGURE 1:**
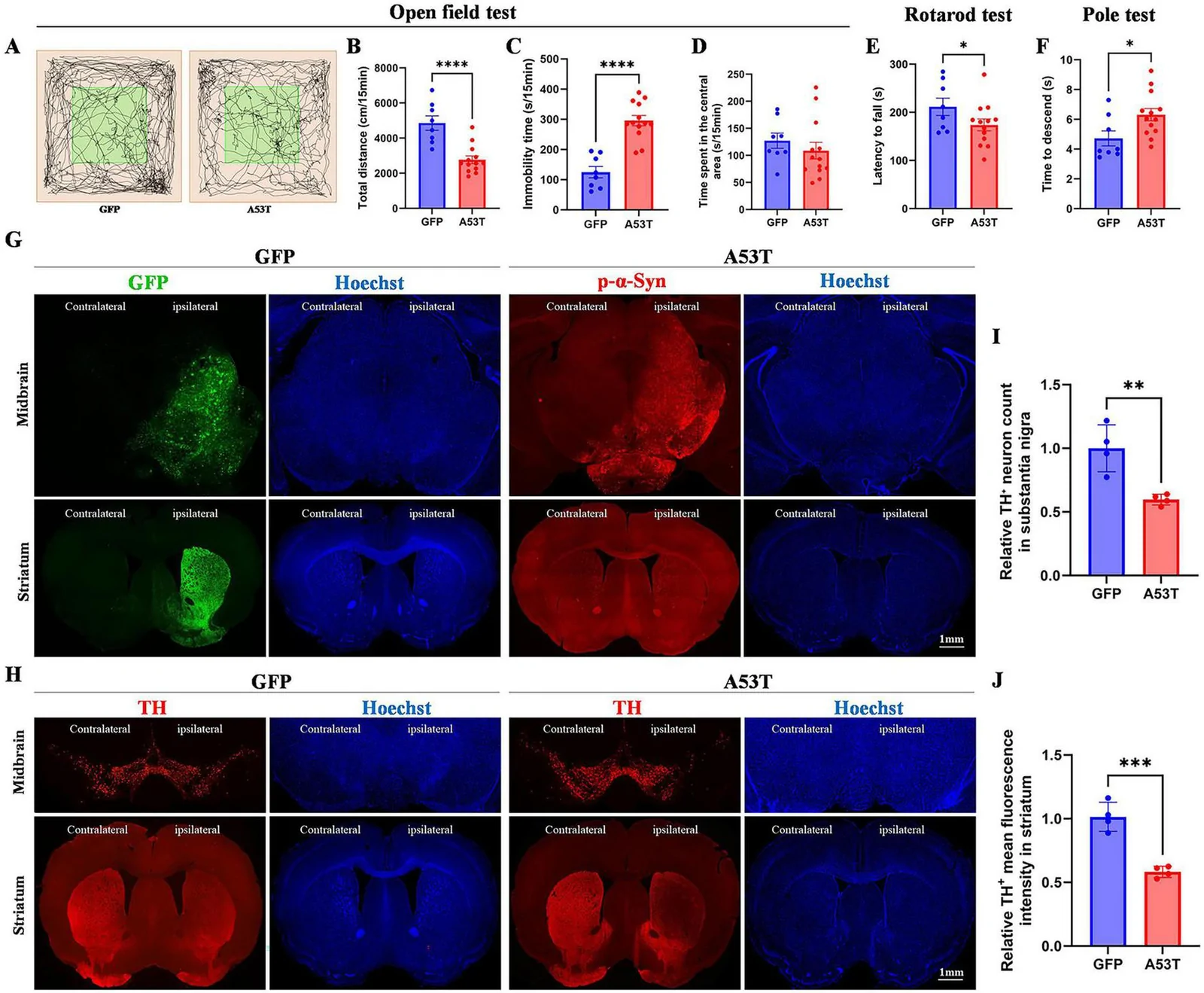
A53T mice exhibit motor dysfunction and loss of dopaminergic neurons in the substantia nigra. **(A–D)** Representative traces, total distance traveled, center zone duration, and immobility time in the open field test at 8 weeks post-injection of AAV9-GFP or AAV9-A53T into the right substantia nigra. **(E)** Latency to fall in the rotarod test. **(F)** Descent time in the pole test. For the GFP and A53T groups, *n* = 8 and 13, respectively. **(G)** Representative immunofluorescence images of phosphorylated α-Synuclein (p-α-Syn) in the substantia nigra and adjacent midbrain areas and striatum of A53T mice, and GFP in the corresponding regions of GFP control mice. Scale bar: 1 mm. **(H)** Representative images of tyrosine hydroxylase (TH) immunostaining in the substantia nigra and striatum. Scale bar: 1 mm. **(I)** Quantification of TH^+^ neurons in the substantia nigra. *n* = 4 mice per group. **(J)** Quantification of TH^+^ fiber density in the striatum. *n* = 4 mice per group. Data are presented as mean ± SEM. Statistical analysis was performed using a two-tailed Student’s *t*-test. *P < 0.05, **P < 0.01, ***P < 0.001, ****P < 0.0001.

### 3.2 Effects of peripheral AAV8-Irisin delivery on motor deficits in A53T mice

To systematically evaluate whether peripheral Irisin delivery ameliorates behavioral deficits, we employed an AAV8 Irisin-Flag vector as previously reported (Islam et al., 2021). This approach has been shown to mediate robust hepatic expression of Irisin and its secretion into the bloodstream, ultimately leading to significantly elevated Irisin levels in the brain following tail vein injection (Islam et al., 2021). The expression and secretion functionality of this vector was confirmed in the present study both *in vitro* and *in vivo* (Supplementary Figure 2). We next asked whether peripherally delivered Irisin could ameliorate motor deficits in the AAV9-A53T model. Mice were divided into four groups: (1) AAV9-GFP + AAV8-GFP (GFP + GFP), (2) AAV9-A53T + AAV8-GFP (A53T + GFP), (3) AAV9-A53T + AAV8-Irisin low dose (1 × 10^10^ vg/mouse; A53T + Irisin-L), and (4) AAV9-A53T + AAV8-Irisin high dose (1 × 10^11^ vg/mouse; A53T + Irisin-H). AAV8 vectors were administered via tail vein injection 2 weeks after stereotaxic injection of AAV9-A53T into the substantia nigra. Body weight and a series of behavioral tests were assessed at baseline (week 0, pre-intervention) and at weeks 2, 4, 6, 10, 14, 18, and 22 post-intervention (Figure 2A). Throughout the 22-weeks experimental period, body weight increased steadily across all groups, with no significant intergroup differences at any time point (Figure 2B), indicating that tail vein injection of AAV8-Irisin had no significant adverse effects on overall growth and nutritional status.

**FIGURE 2:**
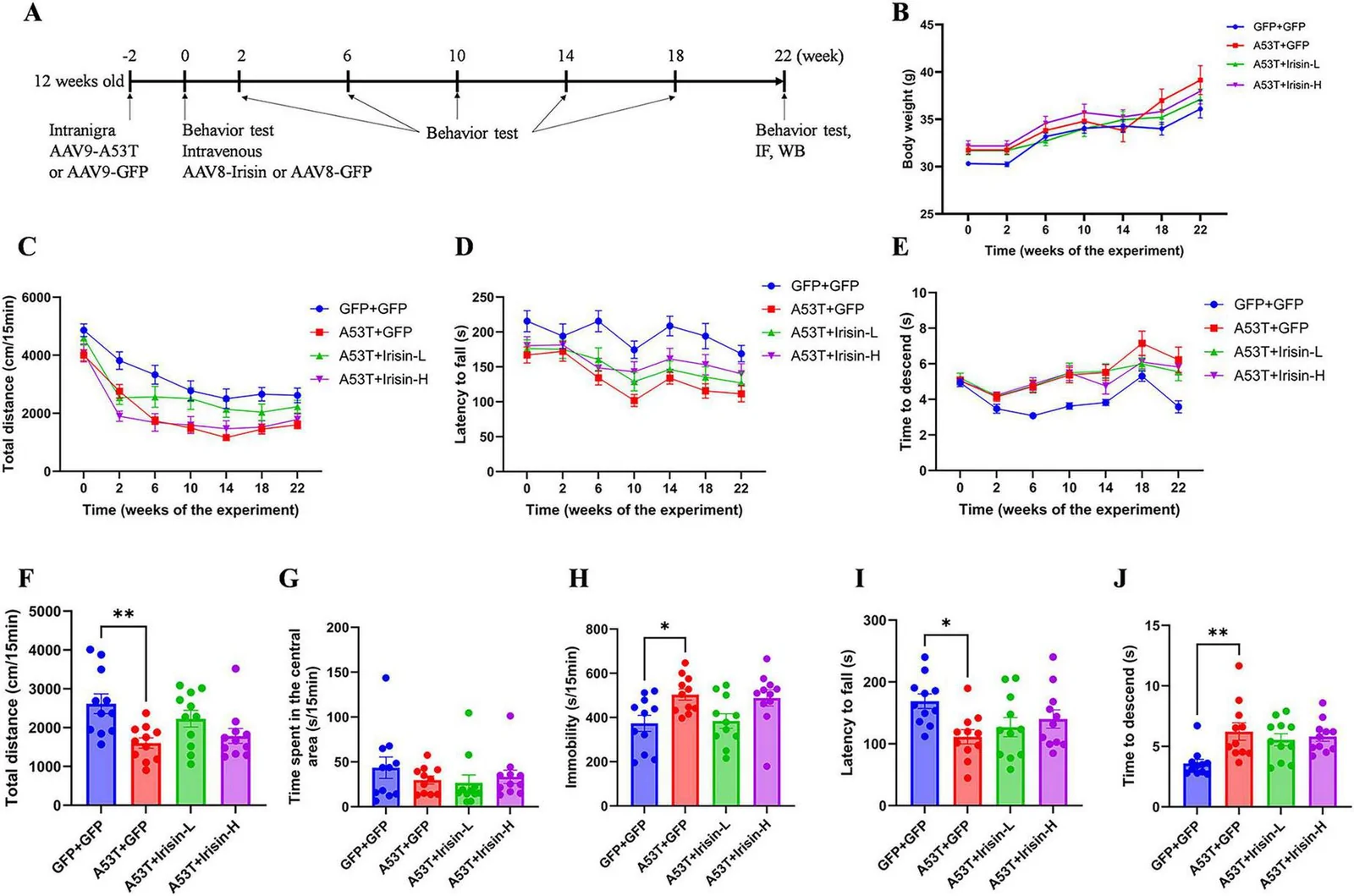
Effects of tail vein-administered AAV8-Irisin on motor deficits in AAV9-A53T mouse model. **(A)** Schematic diagram of the experimental procedure. Twelve-weeks-old mice received an injection of AAV9-A53T into the right substantia nigra for modeling. Two weeks later, AAV8-Irisin (low or high dose) or AAV8-GFP control was injected via the tail vein. Behavioral tests were performed at the indicated time points. **(B)** Body weight curves over the 22-weeks experimental period. **(C)** Temporal profiles of total distance traveled in the open field test. **(D)** Temporal profiles of latency to fall in the rotarod test. **(E)** Temporal profiles of descent time in the pole test. **(F–H)** Quantitative analysis of total distance traveled, center zone duration, and immobility time in the open field test at week 22. **(I)** Quantitative analysis of latency to fall in the rotarod test at week 22. **(J)** Quantitative analysis of descent time in the pole test at week 22. *n* = 11 mice per group. Data are presented as mean ± SEM. For longitudinal assessments **(B–E)**, statistical analysis was performed using two-way repeated-measures ANOVA followed by Šídák’s multiple comparisons test with the A53T+GFP group as the control. Week 22 endpoint data were analyzed by one-way ANOVA followed by Dunnett’s multiple comparisons test with the A53T+GFP group as the control. *P < 0.05, **P < 0.01.

In the open field test, total distance traveled was reduced in A53T + GFP group relative to GFP + GFP controls from week 2 onward. The A53T + Irisin-L group exhibited an increasing trend compared with the A53T + GFP group beginning at week 6 (Figure 2C, Supplementary Figure 3). At the week 22 endpoint, total distance was significantly decreased in the A53T + GFP group compared with GFP + GFP controls. In contrast, neither Irisin-treated group showed a significant increase, despite a modest upward trend in the low-dose group (Figure 2F). Center zone duration did not differ significantly between the A53T + GFP group and any of the other groups (Figure 2G). Immobility time was significantly elevated in the A53T + GFP group compared with GFP + GFP controls. A non-significant trend toward reduced immobility was observed in the low-dose Irisin group, whereas no comparable trend was detected in the high-dose group (Figure 2H). In the rotarod test, latency to fall was shortened in A53T + GFP group relative to GFP + GFP controls from week 6 onward. Although Irisin-treated groups showed a modest prolongation compared with the A53T + GFP group, no statistically significant differences were observed (Figure 2D, Supplementary Figure 4). At the week 22 endpoint, latency was significantly reduced in A53T + GFP group versus GFP + GFP controls, with no appreciable numeric difference between the A53T + GFP and Irisin-treated groups (Figure 2I). In the pole test, descent time was prolonged in A53T + GFP group relative to GFP + GFP controls from week 6 onward. Irisin intervention at either dose did not significantly shorten descent time at any time point (Figure 2E, Supplementary Figure 5). At the week 22 endpoint, descent time remained significantly increased in A53T + GFP group versus GFP + GFP controls, with no appreciable difference among the A53T groups (Figure 2J). In summary, these results showed that peripheral administration of AAV8-Irisin at 2 weeks after the establishment of the A53T mouse model did not significantly improve the behavioral outcomes measured in this study.

### 3.3 Effects of peripheral AAV8-Irisin delivery on dopaminergic neuron loss and α-Synuclein pathology in AAV9-A53T mice

To evaluate the effects of peripheral AAV8-Irisin delivery on dopaminergic neuron survival and α-Synuclein pathology in the substantia nigra of AAV9-A53T mice, immunofluorescence staining was performed to quantify the number of TH^+^ neurons, the level of phosphorylated α-Synuclein (p-α-Syn) deposition in the substantia nigra, and the density of TH^+^ fibers in the striatum. TH protein expression in the striatum was further assessed by Western blotting. Compared with the GFP + GFP control group, A53T + GFP group exhibited a significant reduction in the number of TH^+^ neurons in the injected substantia nigra, accompanied by marked decreases in striatal TH^+^ fiber density and TH protein level. However, relative to the A53T + GFP group, neither the low-dose nor high-dose AAV8-Irisin intervention significantly increased the number of TH^+^ neurons in the substantia nigra (Figures 3A, B). Similarly, neither striatal TH^+^ fiber density nor TH protein expression levels in striatum differed significantly between the A53T+GFP group and the A53T+Irisin groups (Figures 3D–G). Meanwhile, prominent p-α-Syn immunoreactivity was observed in the injected substantia nigra across all A53T groups. Compared with the A53T+GFP group, neither dose of AAV8-Irisin significantly reduced p-α-Syn fluorescence intensity (Figures 3A, C). Overall, these findings suggest that, under the current experimental conditions, tail vein administration of AAV8-Irisin at either low or the high dose did not produce a detectable mitigation of dopaminergic neuron degeneration in the substantia nigra or a reduction in pathological α-Synuclein deposition in A53T mice.

**FIGURE 3:**
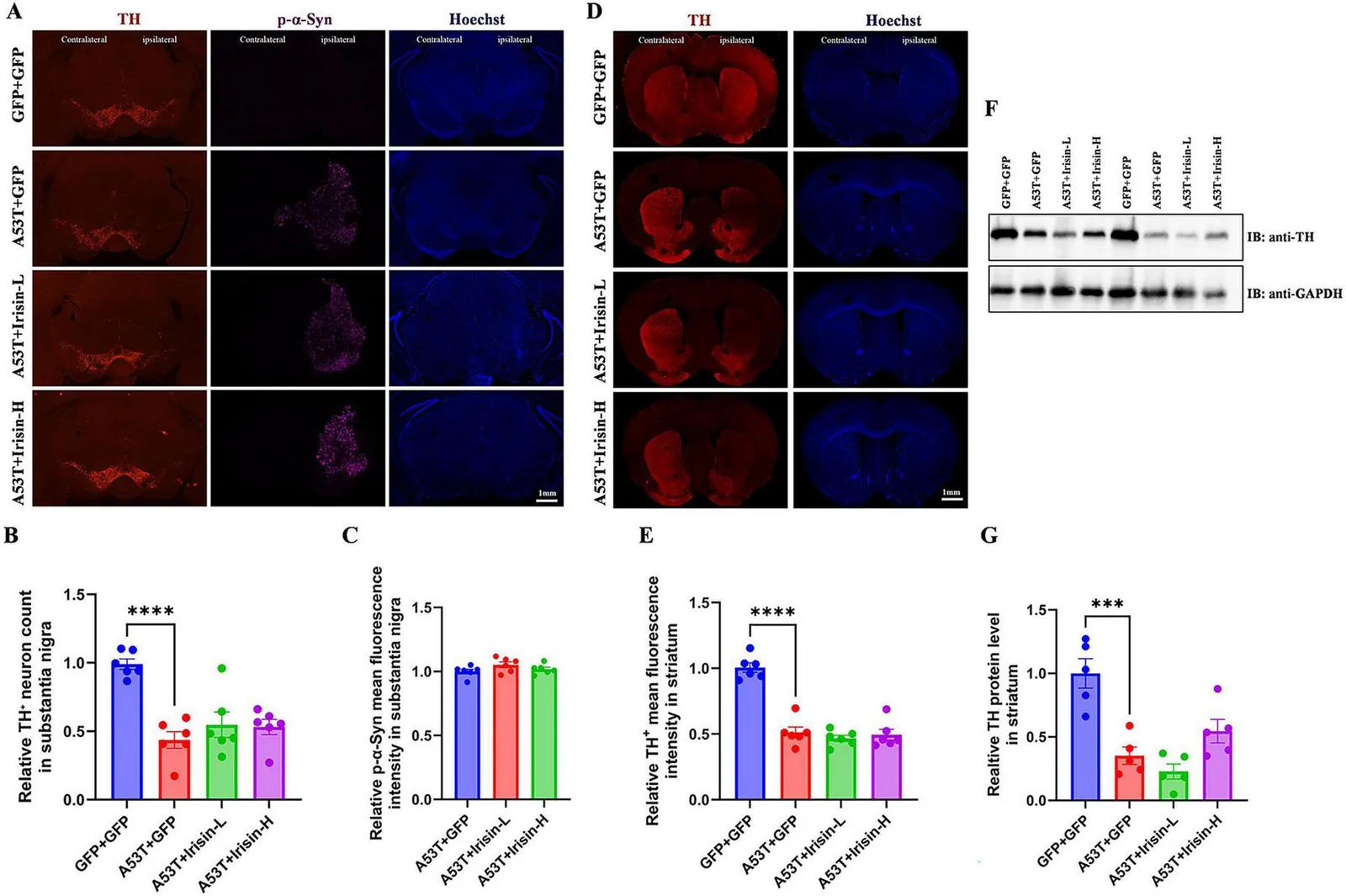
Effects of AAV8-Irisin on dopaminergic neuron loss and α-Synuclein pathology in AAV9-A53T mice. **(A)** Representative TH and p-α-Syn immunofluorescence staining images of the substantia nigra. Scale bar: 1 mm. **(B)** Quantification of TH^+^ neurons in the substantia nigra. *n* = 6 mice per group. **(C)** Quantification of p-α-Syn fluorescence intensity in the substantia nigra. *n* = 6 mice per group. **(D)** Representative TH immunofluorescence staining images of the striatum. Scale bar: 1 mm. **(E)** Quantification of TH^+^ fiber fluorescence intensity in the striatum. *n* = 6 mice per group. **(F)** Representative western blot images of TH protein expression in the striatum. GAPDH served as a loading control. Full blots for all samples are shown in Supplementary Figure 9. **(G)** Quantification of TH protein expression levels in the striatum. *n* = 5 mice per group. Data are presented as mean ± SEM. Statistical analysis was performed using one-way ANOVA followed by Dunnett’s multiple comparisons test with the A53T+GFP group as the control. ***P < 0.001; ****P < 0.0001.

### 3.4 Effects of peripheral AAV8-Irisin delivery on glial activation and AQP4 polarization in AAV9-A53T mice

Previous studies have identified potential Irisin receptors, such as integrin αVβ5, on glial cells in the brain (Islam et al., 2021; Kamidai et al., 2026), suggesting that Irisin may modulate neuroinflammation in Parkinson’s disease. To evaluate whether peripherally administered Irisin influences glial activation in the AAV9-A53T mouse brain, immunofluorescence staining was performed to assess the expression of the astrocyte marker GFAP and the microglial marker IBA1 in the substantia nigra, together with quantitative morphological analysis of astrocytes.

Compared with the GFP+GFP control group, the A53T+GFP group showed significantly higher mean fluorescence intensities of GFAP and IBA1 in the substantia nigra, indicative of pronounced astrocytic and microglial activation. However, relative to the A53T+GFP group, neither low-dose nor high-dose AAV8-Irisin significantly attenuated these elevations (Figures 4A–D). Morphological analysis of astrocytes in the substantia nigra further revealed that total process length, branch complexity, and terminal number were significantly altered in the A53T+GFP group compared with the GFP+GFP control group, representing typical features of reactive astrogliosis. No significant reversal of these morphological changes was observed in the A53T+Irisin groups relative to the A53T+GFP group (Figures 4E–H).

**FIGURE 4:**
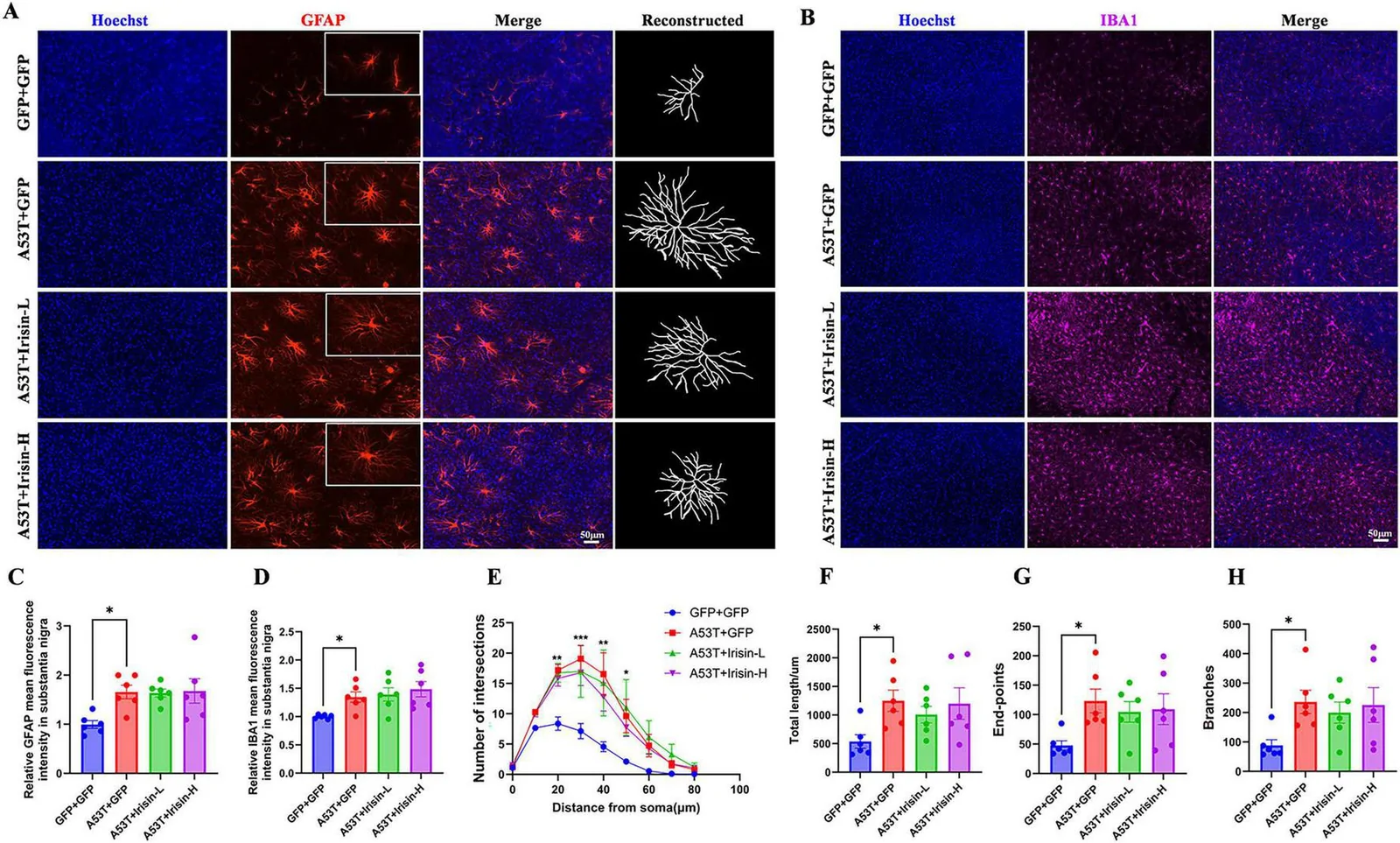
Effects of tail vein-injected AAV8-Irisin on glial cell activation in the substantia nigra of AAV9-A53T mice. **(A)** Representative images of GFAP immunofluorescence staining in the substantia nigra. Scale bar, 50 μm. **(B)** Representative images of IBA1 immunofluorescence staining in the substantia nigra. Scale bar, 50 μm. **(C)** Quantitative analysis of mean GFAP fluorescence intensity in the substantia nigra. *n* = 6 mice per group. **(D)** Quantitative analysis of mean IBA1 fluorescence intensity in the substantia nigra. *n* = 6 mice per group. **(E)** Sholl analysis of astrocyte morphology, showing the number of intersections at different radii. A total of 10–12 astrocytes were analyzed per mouse (*n* = 6 mice per group). **(F)** Quantitative analysis of total astrocyte process length in the substantia nigra. *n* = 6 mice per group. **(G)** Quantitative analysis of astrocyte branch point number in the substantia nigra. *n* = 6 mice per group. **(H)** Quantitative analysis of astrocyte endpoint number in the substantia nigra. *n* = 6 mice per group. Data are presented as mean ± SEM. Statistical analysis was performed using one-way ANOVA followed by Dunnett’s multiple comparisons test with the A53T+GFP group as the control. Brown-Forsythe and Welch ANOVA with Dunnett’s T3 multiple comparisons test was used for panel **(D)**. *P < 0.05, **P < 0.01, ***P < 0.001.

This study also evaluated the effect of Irisin on the polarized distribution of aquaporin-4 (AQP4) in striatal astrocytes, given that perivascular AQP4 polarization is essential for glymphatic clearance of α-Synuclein from the brain. AQP4 immunofluorescence staining revealed a significant reduction in mean AQP4 fluorescence intensity in the striatum of the A53T+GFP group compared with the GFP+GFP group (Figures 5A, B). Quantitative analysis of the AQP4 polarity index further demonstrated that AQP4 polarization was markedly impaired in the A53T+GFP group. Relative to the A53T+GFP group, neither low-dose nor high-dose AAV8-Irisin improved AQP4 expression levels or restored its abnormal polarized distribution in the striatum (Figure 5C). Altogether, these findings suggest that, despite glial expression of the Irisin receptor, tail vein administration of AAV8-Irisin did not produce a detectable suppression of glial activation, reversal of astrocytic morphological remodeling, or restoration of astrocytic AQP4 polarity in AAV9-A53T mice under the current experimental conditions. To further explore whether AAV8-Irisin activated potential neurotrophic or homeostatic signaling pathways under the present experimental conditions, we examined the ERK/BDNF axis, as well as AMPK/SIRT1 signaling and autophagy-related markers. Neither the pERK/ERK ratio nor BDNF protein levels differed significantly between the A53T+GFP and the Irisin-treated groups, suggesting that Irisin did not detectably activate the ERK/BDNF axis in this chronic AAV-A53T model (Supplementary Figure 6). Similarly, peripheral AAV8-Irisin delivery did not significantly alter the p-AMPK/AMPK ratio, SIRT1, p62, or Beclin1 protein levels in A53T mice (Supplementary Figure 7).

**FIGURE 5:**
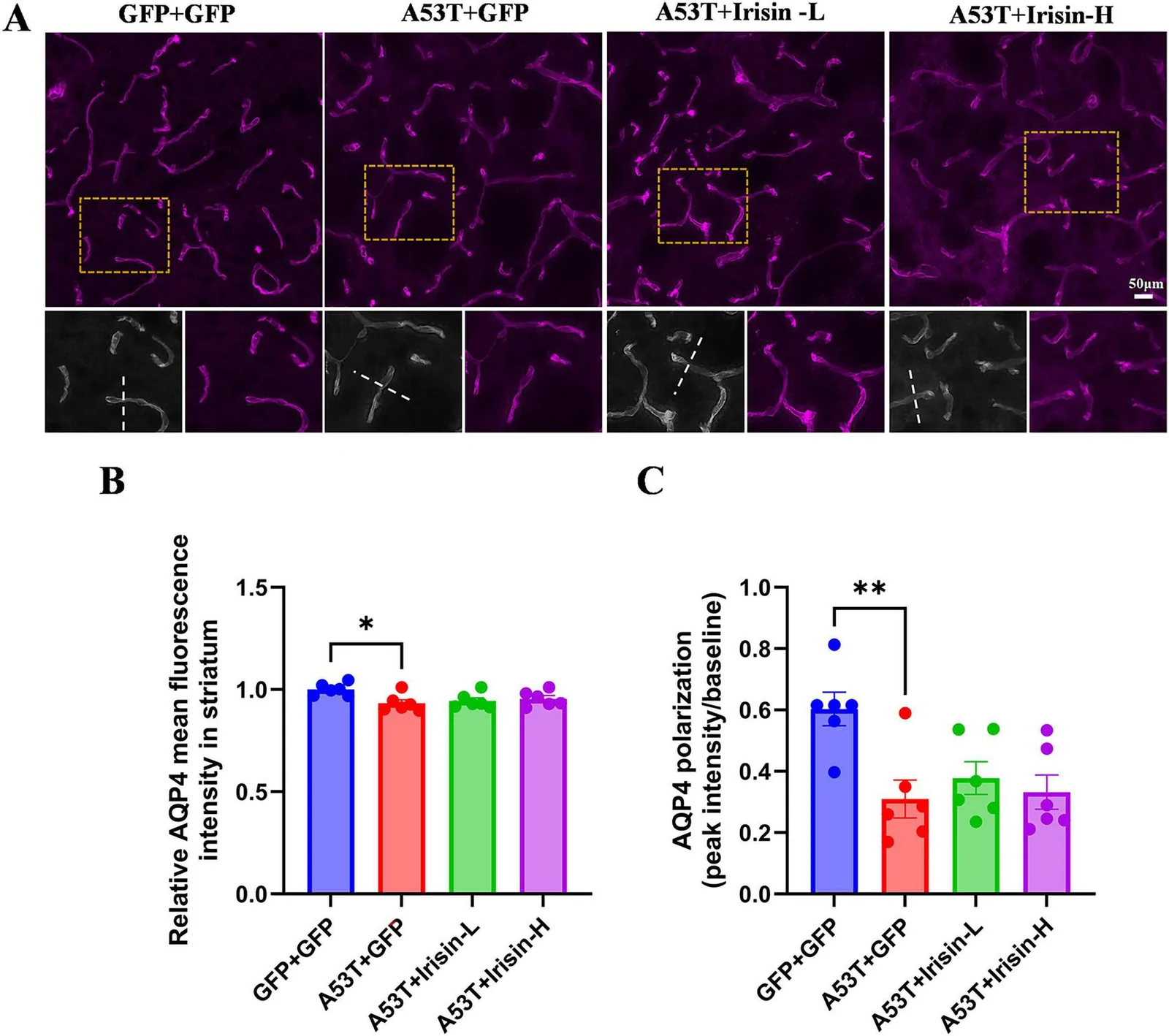
Effects of tail vein-injected AAV8-Irisin on AQP4 polarized distribution in the striatum of AAV9-A53T mice. **(A)** Representative images of AQP4 immunofluorescence staining in the striatum. Scale bar, 50 μm. **(B)** Quantitative analysis of mean AQP4 fluorescence intensity in the striatum. *n* = 6 mice per group. **(C)** Quantitative analysis of the AQP4 polarization (peak intensity/baseline) in the striatum. *n* = 6 mice per group. Data are presented as mean ± SEM. Statistical analysis was performed using one-way ANOVA followed by Dunnett’s multiple comparisons test with the A53T+GFP group as the control. *P < 0.05, **P < 0.01.

### 3.5 Effects of intranigral AAV8-Irisin delivery on motor performance and dopaminergic neuron survival in AAV9-A53T mice

To evaluate the effects of direct intranigral overexpression of Irisin on motor performance and TH^+^ neuron survival in AAV9-A53T mice, AAV9-A53T and AAV8-Irisin were co-delivered into the substantia nigra via stereotaxic injection. Experimental animals were divided into four groups: AAV9-GFP + AAV8-GFP (GFP+GFP), AAV9-GFP + AAV8-Irisin (GFP+Irisin), AAV9-A53T + AAV8-GFP (A53T+GFP), and AAV9-A53T + AAV8-Irisin (A53T+Irisin). Motor coordination and balance were assessed longitudinally using the rotarod test. At week 16, animals were sacrificed to quantify the number of TH^+^ neurons and the level of p-α-Syn deposition in the substantia nigra (Figure 6A). In the rotarod test, latency to fall was shorter in the A53T+GFP group relative to GFP+GFP controls, whereas the A53T+Irisin group exhibited a trend toward prolonged latency compared with the A53T+GFP group (Figure 6B). At the week 16 endpoint, latency was significantly reduced in the A53T+GFP group versus GFP+GFP controls. The A53T+Irisin group showed a numerically longer latency compared with the A53T+GFP group; however, this difference did not reach statistical significance (Figure 6C). Body weight did not differ significantly among groups throughout the experimental period (Figure 6D). Immunofluorescence staining confirmed exogenous Irisin expression in the substantia nigra (Supplementary Figure 8) and revealed a significant reduction in TH^+^ neuron number in the injected substantia nigra of the A53T+GFP group compared with GFP+GFP controls. The A53T+Irisin group showed a numerically higher TH^+^ neuron count relative to the A53T+GFP group, although this difference was not statistically significant. No significant difference in p-α-Syn fluorescence intensity was observed between the A53T+GFP and A53T+Irisin groups (Figures 6E–H). These findings indicate that direct intranigral AAV8-Irisin delivery did not produce statistically significant improvement in either motor performance or dopaminergic neuron survival.

**FIGURE 6:**
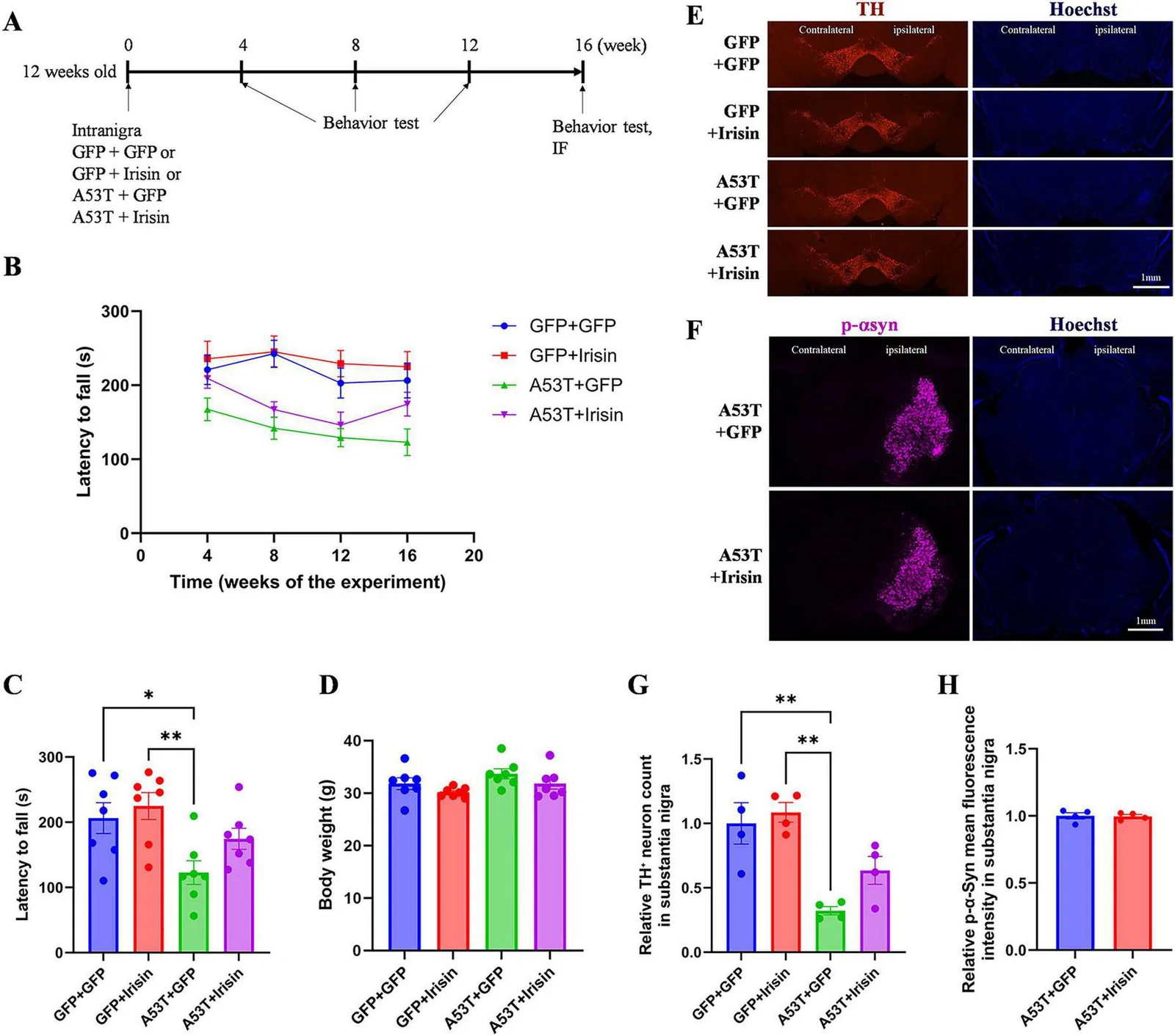
Effects of intranigral AAV8-Irisin delivery on motor performance and dopaminergic neuron survival in AAV9-A53T mice. **(A)** Schematic diagram of the experimental procedure. Twelve-weeks-old mice received stereotaxic co-injections into the substantia nigra and were divided into four groups: GFP+GFP, GFP+Irisin, A53T+GFP, and A53T+Irisin. Behavioral tests were performed at 4, 8, 12, and 16 weeks post-injection. **(B)** Temporal profiles of latency to fall in the rotarod test. *n* = 7 mice per group. **(C)** Quantitative analysis of latency to fall in the rotarod test at week 16. *n* = 7 mice per group. **(D)** Quantitative analysis of body weight at week 16. *n* = 7 mice per group. **(E)** Representative images of TH immunofluorescence staining in the substantia nigra. Scale bar, 1 mm. **(F)** Representative images of p-α-Syn immunofluorescence staining in the substantia nigra. Scale bar, 1 mm. **(G)** Quantitative analysis of TH^+^ neuron count in the substantia nigra. *n* = 4 mice per group. **(H)** Quantitative analysis of p-α-Syn fluorescence intensity in the substantia nigra. *n* = 4 mice per group. Data are presented as mean ± SEM. For longitudinal assessment **(B)**, two-way repeated-measures ANOVA was used. For endpoint measurements **(C,D,G)**, given the 2 × 2 factorial design (A53T × Irisin), two-way ANOVA was applied. All multiple comparisons were corrected using Šídák’s test **(B)** or Tukey’s test **(C,D,G)**. An unpaired two-tailed Student’s *t*-test was used for panel **(H)**. *P < 0.05, **P < 0.01.

## 4 Discussion

In this study, we systematically evaluated the effects of AAV8-mediated peripheral and central delivery of Irisin in the AAV9-A53T mouse model. Neither route of administration significantly ameliorated the core behavioral and neuropathological phenotypes of this model, including motor dysfunction, nigral dopaminergic neuron loss, α-Synuclein pathology, and neuroinflammation. Modest, non-significant improvements were observed under certain conditions. Specifically, the low-dose peripheral group exhibited a numerically lower immobility time at the 22-weeks endpoint, and intranigral co-injection resulted in numerically increased motor performance and TH^+^ neuron counts. Nevertheless, none of these changes reached statistical significance. The absence of a statistically significant therapeutic effect in the AAV-A53T model contrasts with the pronounced neuroprotection conferred by Irisin in the MPTP acute injury model (Cai et al., 2025), the α-Syn preformed fibrils (PFFs) propagation model (Kam et al., 2022), and the 6-hydroxydopamine (6-OHDA)-induced oxidative stress model (Kamidai et al., 2026).

The discrepancy between Irisin’s protective effects in other PD models and its limited effects in the AAV9-A53T model highlights the likely role of pathophysiological heterogeneity in therapeutic outcomes. The AAV9-A53T model drives sustained overexpression of the pathogenic A53T SNCA variant, which causes familial PD. Its core pathological features include a chronic, high-level intracellular α-Syn burden and progressive proteostatic collapse (Ip et al., 2017; Oliveras-Salvá et al., 2013; Sato et al., 2011). Pathological changes in this model are largely cell-autonomous. In contrast, the MPTP model mimics selective dopaminergic neuron necrosis triggered by acute mitochondrial intoxication, characterized by intense but transient inflammation and prominent mitochondrial dysfunction (Beal, 2003). Irisin effectively alleviates MPTP-induced injury by activating the AMPK/SIRT1 signaling axis, thereby restoring mitochondrial bioenergetics and suppressing oxidative stress (Cai et al., 2025; Li et al., 2025). In the PFFs model, pathological progression is driven primarily by the prion-like intercellular spread of misfolded α-Syn (Uemura, 2025; Wu and Schekman, 2024). Recent evidence indicates that Irisin enhances autophagic flux and promotes the endolysosomal degradation of pathological α-Syn, thereby accelerating the clearance of α-Syn monomers before they are incorporated into aggregate cores (Kam et al., 2022; Cai et al., 2026; Zhu et al., 2025). In the present study, peripheral AAV8-Irisin delivery did not significantly alter the pAMPK/AMPK ratio, SIRT1, p62, or Beclin1 protein levels in the striatum. Together, these comparisons raise the possibility that the protective effects of Irisin differ across pathological contexts; however, whether such differences reflect genuine model-dependent efficacy remains to be determined through direct head-to-head comparisons.

Neuroinflammation, characterized by reactive astrogliosis and microglial activation, is a prominent pathological feature of Parkinson’s disease and is thought to contribute to dopaminergic neurodegeneration. In the MPTP model, Irisin has been reported to attenuate glial activation, suggesting that anti-inflammatory effects may contribute to its neuroprotective actions (Cai et al., 2025). In the present study, however, Irisin did not produce a detectable suppression of astrocytic and microglial activation in the substantia nigra of A53T mice. This discrepancy may reflect the distinct dynamics of neuroinflammation in chronic versus acute disease settings (Badr et al., 2022; Hébert et al., 2003; Li et al., 2021). Recent single-cell transcriptomic analyses have shown that glial cells undergo progressive phenotypic transitions during α-Syn pathology, shifting from early homeostatic state toward late, pro-inflammatory phenotypes (Patani et al., 2023; Zhang et al., 2025), raising the possibility that glial cells had already entered a chronic, self-sustaining activation state by the time of Irisin intervention. It is important to underscore, however, that our assessment of neuroinflammation was confined to IBA1 and GFAP immunoreactivity and astrocyte morphological analysis, which capture broad changes in glial reactivity but cannot resolve specific molecular pathways. Whether the absence of a detectable anti-inflammatory effect stems from an inability of Irisin to engage its putative targets in this model, or from the presence of persistent or altered glial states (Chowdhury et al., 2025), remains to be determined. Likewise, whether specific inflammatory mediators such as the NLRP3 inflammasome or downstream cytokines contribute to the observed lack of anti-inflammatory effect cannot be inferred from our data. Thus, while these results suggest that Irisin, under the tested regimen, does not attenuate broad neuroinflammatory responses in this chronic model, the underlying mechanistic explanations remain speculative and warrant direct molecular investigation in future studies.

This study also examined the effect of Irisin on the polarized distribution of aquaporin-4 (AQP4) in the AAV9-A53T model. AQP4, the predominant water channel in the brain, is primarily localized to perivascular astrocytic endfeet, and its polarized expression is essential for maintaining glymphatic clearance of interstitial solutes, including pathological proteins such as α-Synuclein. In PD, disrupted AQP4 polarity is recognized as a key molecular event underlying impaired glymphatic clearance, as confirmed in both patients and animal models (Lapshina and Ekimova, 2024; Lopes et al., 2025), and loss of this polarity has been shown to exacerbate α-Syn pathology (Yue et al., 2024). In the A53T mice examined here, AQP4 polarization in the striatum was markedly impaired compared with control animals, consistent with previous reports (Zhang et al., 2023). Peripheral Irisin delivery did not detectably restore the impaired polarized distribution of AQP4 in the striatum of A53T mice. Consistent with the absence of significant behavioral and neuropathological protection, these findings indicate that AQP4 polarity was not detectably improved by Irisin under the current experimental conditions.

Even when the blood-brain barrier limitation was bypassed using a co-injection strategy directly into the substantia nigra, the protective effects of Irisin remained limited. This observation raises questions about the delivery modality. The AAV-mediated expression pattern used in this study produces sustained, continuous Irisin exposure, which differs fundamentally from the intermittent, pulsatile release of Irisin that occurs during exercise (Huh et al., 2015). Under physiological conditions, exercise-induced Irisin is released in a transient, activity-dependent manner, and such intermittent stimulation may be more effective at activating downstream protective pathways than sustained ligand exposure (Storlino et al., 2020). Whether the pharmacokinetic differences between continuous AAV-mediated delivery and episodic physiological release affect receptor activation, downstream signaling, or overall neuroprotective efficacy was not directly assessed in the present study. This remains an important consideration for future investigations, which may benefit from delivery strategies that more closely mimic the endogenous dynamics of Irisin release.

Beyond these design-related considerations, several model-related and translational factors further limit the interpretation of our findings. Fourth, the use of AAV-GFP as a control vector may have introduced an unintended confound. It has been reported that AAV-mediated GFP expression itself can cause toxicity and dopaminergic neuron loss in the substantia nigra, with effects comparable in magnitude to those induced by α-Synuclein overexpression (Negrini et al., 2022). Such background toxicity could partially mask a subtle protective effect of Irisin, and reliance on a GFP control group may therefore dilute the ability to detect modest benefits. We therefore recommend that future studies incorporate an empty-vector or non-toxic reporter control to fully resolve this issue. Beyond viral vector-related artifacts, the translational gap between rodent models and human Parkinson’s disease is further compounded by differences in muscle atrophy phenotypes, neuroinflammatory kinetics, and neuromuscular junction integrity, which may collectively influence the responsiveness to Irisin (Zhang et al., 2024). Fifth, only male mice were used, which precludes assessment of potential sex-dependent differences in Irisin responsiveness. Sixth, as Irisin functions as a myokine mediating muscle-brain crosstalk, its neuroprotective efficacy may be modulated by concurrent skeletal muscle atrophy-a common comorbidity in advanced neurodegenerative diseases that can disrupt systemic metabolic and inflammatory signaling (Zhang et al., 2026). Given that the present study did not assess skeletal muscle status, we cannot exclude the possibility that subclinical muscle dysfunction influenced the bioavailability or downstream effects of exogenous Irisin. Finally, it should be noted that the behavioral assessments employed in this study-open field, rotarod, and pole tests-are relatively coarse measures of global motor function. In a unilateral nigral lesion model such as the AAV-A53T model, these tests may not be optimally sensitive to subtle or lateralized motor deficits. It is therefore possible that a real functional improvement could have been masked by the use of whole-body locomotor readouts that do not distinguish between the ipsilateral and contralateral sides. Future studies might consider incorporating more sensitive and side-specific assessments, such as gait analysis or the cylinder test, to better capture partial or lateralized functional recovery.

An additional limitation is the relatively small sample size for several histological and biochemical endpoints. No formal *a priori* power calculation was performed for these exploratory experiments. Although several robust pathological differences between AAV-GFP and AAV-A53T mice were detected with the current sample sizes, these numbers may not have provided adequate statistical power to detect smaller treatment effects of Irisin. Therefore, the absence of statistically significant differences should not be interpreted as definitive evidence of no biological effect. Larger, prospectively powered studies will be required to determine whether Irisin produces any neuroprotective effects in this model.

In summary, under the present experimental conditions, neither peripheral AAV8-mediated Irisin delivery nor direct intranigral AAV8-Irisin co-administration produced statistically significant neuroprotective effects in the AAV9-A53T α-Synuclein overexpression model. Whether the efficacy of Irisin varies across disease models, pathological stages, or delivery kinetics cannot be determined from this single-study design and requires dedicated head-to-head comparisons. Our findings do not exclude the possibility that Irisin-mediated protection could be achieved under different pathological contexts or with alternative delivery strategies; future studies designed to directly test these variables will be necessary to clarify the therapeutic potential of Irisin in PD.

## Data Availability

The original contributions presented in this study are included in this article/Supplementary material, further inquiries can be directed to the corresponding authors.
