# Supplementary material for: Evaluation of peripheral and intranigral AAV-mediated Irisin delivery in a chronic AAV-A53T α-Synuclein overexpression mouse model of Parkinson’s disease
Source: Front Aging Neurosci. 2026 Sep 28;18:1918326. doi: 10.3389/fnagi.2026.1918326 (PMC13655144; doi:10.3389/fnagi.2026.1918326)
Supplement: Supplementary file 1 [file Data_Sheet_1.docx]

Supplementary Material

## 1.1 Supplementary Figures

**Supplementary Figure 1.** **Immunofluorescence of the substantia nigra and striatum in AAV9-A53T injected mice. (A)** Co-staining of p-α-Syn (purple), TH (red), and Hoechst (blue) in the substantia nigra. Scale bar: 100 μm. **(B)** Flag (detecting exogenous A53T hα-Syn) and TH (dopaminergic fibers) staining in the ipsilateral striatum. Scale bar: 1 mm.

**Supplementary Figure** 2. Validation of AAV8-Irisin-mediated expression in vitro and in vivo**.** (A) HEK293 cells were transfected with an AAV8 backbone expression plasmid encoding Irisin or a control vector. Cell lysates and culture supernatants were collected and analyzed by immunoblotting with anti-Flag, anti-FNDC5, and anti-GAPDH antibodies. Irisin-specific bands were detected in both lysates and supernatants from Irisin-transfected cells, whereas no corresponding signals were observed in the control group. (B) Wild-type mice received tail vein injections of AAV8-GFP (GFP) or AAV8-Irisin at low (1×10¹⁰ vg/mouse) or high (1×10¹¹ vg/mouse) titer. RT-PCR analysis of liver tissues collected 6 weeks post-injection confirmed Irisin mRNA expression in both AAV8-Irisin-injected groups. (C) Quantitative RT-PCR analysis of Irisin mRNA levels in liver tissues from the indicated groups. Irisin mRNA expression was significantly upregulated in both low- and high-dose AAV8-Irisin groups compared with EV controls. n = 4 mice per group. Data are presented as mean ± SEM. Statistical analysis was performed using one-way ANOVA by Dunnett's multiple comparisons test with the GFP group as the control. **P < 0.01.

**Supplementary Figure 3. Effects of AAV8-Irisin on open field test performance in AAV9-A53T mice.** Quantitative analysis of total distance traveled at weeks 0 (A), 2 (B), 6 (C), 10 (D), 14 (E), and 18 (F) post-injection. n = 11 mice per group. Data are shown as mean ± SEM. Statistical analysis was performed using one-way ANOVA by Dunnett's multiple comparisons test with the A53T+GFP group as the control. Brown-Forsythe and Welch ANOVA with Dunnett’s T3 multiple comparisons test was used for (E). *P < 0.05, **P < 0.01.

**Supplementary Figure 4. Effects of AAV8-Irisin on rotarod test performance in AAV9-A53T mice.** Quantitative analysis of latency to fall at weeks 0 (A), 2 (B), 6 (C), 10 (D), 14 (E), and 18 (F) post-injection. n = 11 mice per group. Data are shown as mean ± SEM. Statistical analysis was performed using one-way ANOVA by Dunnett's multiple comparisons test with the A53T+GFP group as the control. *P < 0.05, **P < 0.01, ***P < 0.001.

**Supplementary Figure 5. Effects of AAV8-Irisin on pole test performance in AAV9-A53T mice.** Quantitative analysis of time to descend at weeks 0 (A), 2 (B), 6 (C), 10 (D), 14 (E), and 18 (F) post-injection. n = 11 mice per group. Data are shown as mean ± SEM. Statistical analysis was performed using one-way ANOVA by Dunnett's multiple comparisons test with the A53T+GFP group as the control. *P < 0.05, **P < 0.01.

**Supplementary Figure 6. Effects of peripheral AAV8-Irisin delivery on BDNF/ERK signaling in AAV9-A53T mice.** Western blot analysis of pERK, total ERK, and BDNF expression in striatal tissues. Quantification showed no significant differences in pERK/ERK ratio or BDNF protein levels among A53T+GFP, A53T+Irisin-L, and A53T+Irisin-H groups. Data are presented as mean ± SEM. Statistical analysis was performed using one-way ANOVA by Dunnett's multiple comparisons test with the A53T+GFP group as the control.

**Supplementary Figure 7. Effects of AAV8-Irisin on AMPK signaling and autophagy-related proteins in the striatum of AAV9-A53T mice.** Western blot analysis of SIRT1, pAMPK, AMPK, P62, and Beclin1expression in the striatum. Quantification showed no significant differences in relative SIRT1 **(B)**, pAMPK/AMPK ratio **(C)**, P62 **(D)**, Beclin1 **(E)** protein levels among A53T+GFP, A53T+Irisin-L, and A53T+Irisin-H groups. Data are presented as mean ± SEM. Statistical analysis was performed using one-way ANOVA by Dunnett's multiple comparisons test with the A53T+GFP group as the control.

**Supplementary Figure 8. Immunofluorescence detection of exogenous Irisin in the substantia nigra.** Representative immunofluorescence images of the injected substantia nigra from GFP+Irisin mice. Exogenous Irisin was detected using an anti-Flag antibody, and dopaminergic neurons were labeled with an anti-TH antibody. Scale bar, 100 μm.

**Supplementary Figure S9**. **The original image of Western Blot.** Uncropped Western blots for Fig. 4F are provided, with the bands used in the final figure indicated by square boxes.

## 1.2 Supplementary Materials and Methods

**Cell culture and transfection**

HEK293 cells were cultured in high-glucose Dulbecco's Modified Eagle Medium (DMEM) supplemented with 10% fetal bovine serum (FBS) and 1% penicillin-streptomycin. The cells were maintained in a humidified incubator at 37 °C with 5% CO₂. For transfection, HEK293 cells in logarithmic growth phase were harvested by trypsinization, counted, and seeded into 6-well plates approximately 24 hours before transfection. Seeding was performed using antibiotic-free complete medium to achieve 70-80% confluence on the day of transfection. The expression vector encoding Irisin-Flag was extracted using an endotoxin-free plasmid kit (Tiangen Biotech, DP108). Transfection was carried out with Lipofectamine 3000 (Invitrogen, L3000001) following the manufacturer’s instructions. At 48 hours post-transfection, both cell lysates and culture supernatants were collected. The culture supernatants were subsequently concentrated using Ultra Centrifugal Filter units (Merck, UFC501024).

**RNA extraction and real-time PCR**

Total RNA was extracted from mouse liver tissues using RNAiso Plus (Takara, 9108) according to the manufacturer's instructions. Reverse transcription was subsequently performed using the PrimeScript RT Reagent Kit (Takara, RR037Q) to synthesize complementary DNA (cDNA). The mRNA expression levels of *Irisin* and the internal control *Gapdh* were quantified by real-time PCR using TB Green Premix Ex Taq (Takara, RR420A). The primer sequences for *Irisin* were as follows: Forward, 5′-CTGGAGGATGAAGTGGTCATTG-3′; Reverse, 5′-TGGTGTTCACCTCCTGAATG-3′. Following normalization to *Gapdh* expression, the relative Irisin expression was calculated using the 2⁻^ΔΔCt^ method. All reactions were performed in triplicate.

**Antibodies used in Western blotting**

The primary antibodies used for Western blotting were as follows: anti-ERK (Cell Signaling Technology, 9102; 1:1000), anti-phospho-ERK (Thr202/Tyr204) (Cell Signaling Technology, 9101; 1:1000), anti-BDNF (Abclonal, A18129; 1:10000), anti-GAPDH (Proteintech, 81640-5-RR; 1:10000), anti-AMPK (Cell Signaling Technology, 5831; 1:1000), anti-phospho-AMPK (Thr172) (Cell Signaling Technology, 2535; 1:1000), anti-SIRT1 (Abcam, ab110304; 1:1000), anti-Beclin 1 (Cell Signaling Technology, 3495; 1:1000), and anti-p62 (proteintech, 18420-1-AP; 1:10000).
